# Radiotherapy Modality and Primary Tumor Type as Determinants of Outcomes in Brain Metastases: A Retrospective Study of 734 Patients

**DOI:** 10.3390/medicina62091665

**Published:** 2026-08-30

**Authors:** Lucie Hnidakova, Andrea Pagacova, Sarka Kristkova, Jan Svancara, Ludmila Hynkova, Petr Pospisil, Renata Belanova, Jana Maistryszinova, Radek Lakomy, Tomas Prochazka, Miroslav Vrzal, Jiri Sana, Radim Jancalek, Martin Smrcka, Ivana Kolouskova, Lukas Moran, Katerina Prochazkova, Vera Andraskova, Jan Garcic, Pavel Slampa, Pavel Fadrus, Tomas Kazda

**Affiliations:** 1 Department of Radiation Oncology, Masaryk Memorial Cancer Institute, Zluty kopec 7, Brno 65653, Czech Republic; lucie.hnidakova@mou.cz (L.H.);; 2 Department of Radiation Oncology, Faculty of Medicine, Masaryk University, Kamenice 753/5, Brno 62500, Czech Republic; 3 Department of Health Information, Masaryk Memorial Cancer Institute, Zluty kopec 7, Brno 65653, Czech Republic; 4 Department of Medical Imaging, Masaryk Memorial Cancer Institute, Zluty kopec 7, Brno 65653, Czech Republic; 5 Department of Comprehensive Cancer Care, Masaryk Memorial Cancer Institute, Zluty kopec 7, Brno 65653, Czech Republicivana.kolouskova@mou.cz (I.K.); 6 Department of Comprehensive Cancer Care, Faculty of Medicine, Masaryk University, Kamenice 753/5, Brno 65653, Czech Republic; 7 Central European Institute of Technology, Masaryk University, Zerotinovo namesti 617/9, Brno 60177, Czech Republic; 8 Department of Neurosurgery, St. Anne’s University Hospital Brno, Pekarska 664/53, Brno 60200, Czech Republic; 9 Department of Neurosurgery, St. Anne’s University Hospital Brno, Faculty of Medicine, Masaryk University, Kamenice 753/5, Brno 62500, Czech Republic; 10 Department of Neurosurgery, University Hospital Brno, Jihlavska 20, Brno 62500, Czech Republic; 11 Department of Neurosurgery, Faculty of Medicine, Masaryk University, Kamenice 753/5, Brno 62500, Czech Republic; 12 Research Centre for Applied Molecular Oncology (RECAMO), Masaryk Memorial Cancer Institute, Zluty kopec 7, Brno 65653, Czech Republic; 13 Department of Histology and Embryology, Faculty of Medicine, Masaryk University, Kamenice 3, Brno 62500, Czech Republic; 14 Cinical Nutrition Unit, Masaryk Memorial Cancer Institute, Zluty kopec 7, Brno 65653, Czech Republic

**Keywords:** radiotherapy, brain metastases, whole-brain radiotherapy, stereotactic radiosurgery, stereotactic radiotherapy

## Abstract

*Background and Objectives*: Radiotherapy (RT) is a standard component of palliative treatment for brain metastases. The aim of this retrospective analysis of consecutive patients treated between 2017 and 2023 is to analyze overall survival after brain radiotherapy depending on the type of radiotherapy delivered and to monitor the shift from whole-brain radiotherapy (WBRT) to stereotactic radiotherapy (SRS/FSRT) in real-world practice before the era of routinely used modern brain-penetrant targeted therapy. *Materials and Methods*: A total of 734 patients treated with radiotherapy for brain metastases were analyzed. Clinical data were collected via an electronic hospital information system and radiotherapy details were obtained using the Eclipse™ treatment planning system. The survival analysis divides patients according to cancer types and according to the RT technique used. *Results*: A total of 734 patients (median age 65.2 years; 50.5% women) were analyzed, with 104 undergoing more than one course of radiotherapy. Lung cancer (42.3%) and breast cancer (17.2%) were the most common primary diagnoses. Median overall survival (OS) after radiotherapy for the entire cohort was 2.8 months (2.6–3.2 months). Survival varied by radiotherapy technique: 2 months for WBRT-based approaches, 5.7 months for stereotactic radiotherapy (SRS/FSRT), and 7 months for combined WBRT and stereotactic treatment. Breast cancer patients had the longest median OS (3.6 months), with substantially improved outcomes in those treated with stereotactic or combined approaches (12.8 and 11.9 months, respectively). A decline in treated patients was observed during the COVID-19 pandemic (notably in 2021) and a consistent increase in the use of stereotactic techniques between 2017 and 2023 was noted. *Conclusions*: A wide range of radiotherapy techniques are used in the treatment of brain metastases with the primary concern being the prognosis and diagnosis of a patient. A clearly identifiable trend is a shift away from WBRT toward an increased use of targeted stereotactic irradiation techniques for brain lesions. Overall survival varies significantly across primary sites with most favorable outcomes in breast cancer patients irradiated with SRS/FSRT.

## 1. Introduction

Secondary brain tumors are the most common type of intracranial malignancies among adults, affecting approximately one third of patients with cancer. Their incidence continues to rise, partly reflecting improved systemic disease control and prolonged patient survival. Compared with primary brain tumors, intracranial metastases are ten times more frequent [1], making them a major clinical burden in radiation oncology. Treatment approaches may also vary according to the technical resources available at an individual institution, particularly with regard to imaging, delivery platforms, and dedicated planning software.

The treatment of brain metastases (BM) requires a complex and individualized approach [2]. The main options include neurosurgery, often used for accessible, solitary or symptomatic lesions, and radiotherapy (RT), which involves whole-brain radiotherapy (WBRT), stereotactic radiotherapy (SRT), and stereotactic radiosurgery (SRS) [3]. In recent years, the range of RT techniques has expanded to include whole-brain radiotherapy with hippocampal avoidance (HA-WBRT) [4]. Moreover, the possibility of simultaneous dose boosting (SIB) to metastatic lesions, without increasing the overall treatment time by adding additional boost fractions after completion of the standard treatment, enables the hippocampal avoidance of whole-brain radiotherapy with SIB (HA-WBRT- SIB). The aim of these techniques is to first and foremost reduce the likelihood of iatrogenic cognitive impairment and to preserve quality of life [5,6].

Advances in systemic treatment, including targeted therapy and immunotherapy, have led to the development of agents capable of crossing the blood–brain barrier due to their specific size and molecular characteristics. This barrier protects the brain but has until now limited the penetration of systemic therapeutics into the central nervous system [7,8]. Although this article focuses on radiotherapy for brain metastases, it should be emphasized that radiotherapy represents only one component of a comprehensive multimodal treatment strategy, in which modern systemic therapies play an increasingly important role.

Despite general advances in modern oncology, the prognosis for patients with BM remains dismal, with median overall survival typically measured in months. However, some patients may live for several years with brain metastases, with a good quality of life, thanks to combined oncological treatment [9,10]. Importantly, even in these long-term survivors, the treatment of BM remains primarily palliative in nature, and as survival improves, preserving quality of life and neurocognitive function becomes increasingly important, particularly given the potential long-term consequences of intracranial treatment [11].

In this context, institutional treatment patterns and technological evolution play a crucial role in shaping clinical practice. A comprehensive understanding of local patient characteristics and radiotherapy strategies is essential to establish a reference framework for future outcome-driven analyses. Therefore, the aim of this retrospective single-institution study is to characterize the baseline demographic and clinical profile of patients treated for BM at our center and to evaluate temporal trends in the utilization of conventional and advanced radiotherapy techniques. By mapping changes in treatment paradigms over time, we seek to provide an institutional benchmark and a foundation for subsequent hypothesis-driven investigations.

## 2. Materials and Methods

### 2.1. Patient Selection and Radiotherapy

A consecutive series of patients with BM referred to our tertiary comprehensive cancer center for brain radiotherapy between 2017 and 2023 was screened. Patients deemed ineligible for radiotherapy, e.g., due to poor performance status, as well as those who declined radiotherapy, were excluded from this analysis. The clinical data collected from the hospital information system included the primary tumor type, the date of initiation and completion of radiotherapy and the date of the last follow-up or the date of death.

The Eclipse^TM^ (Varian medical systems, Palo Alto, CA, USA) treatment planning system was harvested to obtain the basic RT data including the number of fractions, the dose per fraction, the plan name, the planning target volume (PTV) size, the beam energy, and the planning technique. Based on these parameters, it was possible to distinguish the following radiotherapy techniques: WBRT (whole-brain radiotherapy) planned in 2D using an RT simulator, WBRT planned in 3D using a CT simulation, WBRT-SIB (simultaneous integrated boost), HA-WBRT, HA-WBRT-SIB, SRS (stereotactic radiosurgery), and FSRT (fractionated stereotactic radiotherapy).

The survival analysis divides patients according to cancer types and according to the RT technique used as follows: Group 1—patients with WBRT (includes patients with WBRT CT and RT simulator planning and special WBRT techniques; HA-WBRT, HA-WBRT-SIB), Group 2—SRS/FSRT (one or more courses of SRS/FSRT) and Group 3—WBRT + SRS/FSRT. A total number of 734 patients irradiated from 2017 to 2023 were analyzed; during this time period, 104 patients underwent more than one course of radiotherapy.

### 2.2. Statistical Analysis

Overall survival (OS) was calculated from the date of initiation of radiotherapy to death from any cause. Patients who were alive at the time of the last available follow-up were censored at the date of their last follow-up. Categorical variables were summarized using absolute and relative frequencies, whereas continuous variables were described using the median and range (minimum–maximum). Survival data were analyzed using standard time-to-event methods, with censoring applied to patients who had not experienced the event of interest at the end of the observation period.

Survival probabilities were estimated using the Kaplan–Meier method, and median OS was reported with corresponding 95% confidence intervals (CIs). Kaplan–Meier curves were used to visualize survival outcomes for the entire cohort and according to radiotherapy technique and primary tumor type. Median follow-up time was estimated using the reverse Kaplan–Meier method [12], in which the event indicator is reversed so that censored observations are treated as events. The 95% confidence interval was derived from the Kaplan–Meier confidence interval of the survival function.

All statistical analyses were performed using the Python programming language, version 3.11. Data management, statistical calculations, survival analyses, and graphical visualization were performed using the pandas, numpy, lifelines, plotly, and scipy libraries. The analysis was conducted using standard statistical procedures appropriate for descriptive and time-to-event data. Statistical results are summarized and presented in a manner suitable for clinical interpretation.

## 3. Results

### 3.1. Patient Characteristics

A total of 734 patients were included in the analysis. During the study period, 104 patients underwent more than one course or radiotherapy. Of the entire cohort, 371 (50.5%) were women. The median age at the start of radiotherapy was 65.2 years, range 23.8–86.7 years. The most common primary diagnosis was lung cancer, accounting for (311; 42.3%), followed by breast cancer (126; 17.2%). Basic clinical characteristics and distribution of individual tumors are summarized in Table 1.

### 3.2. Overall Survival After RT

**Figure 1:**
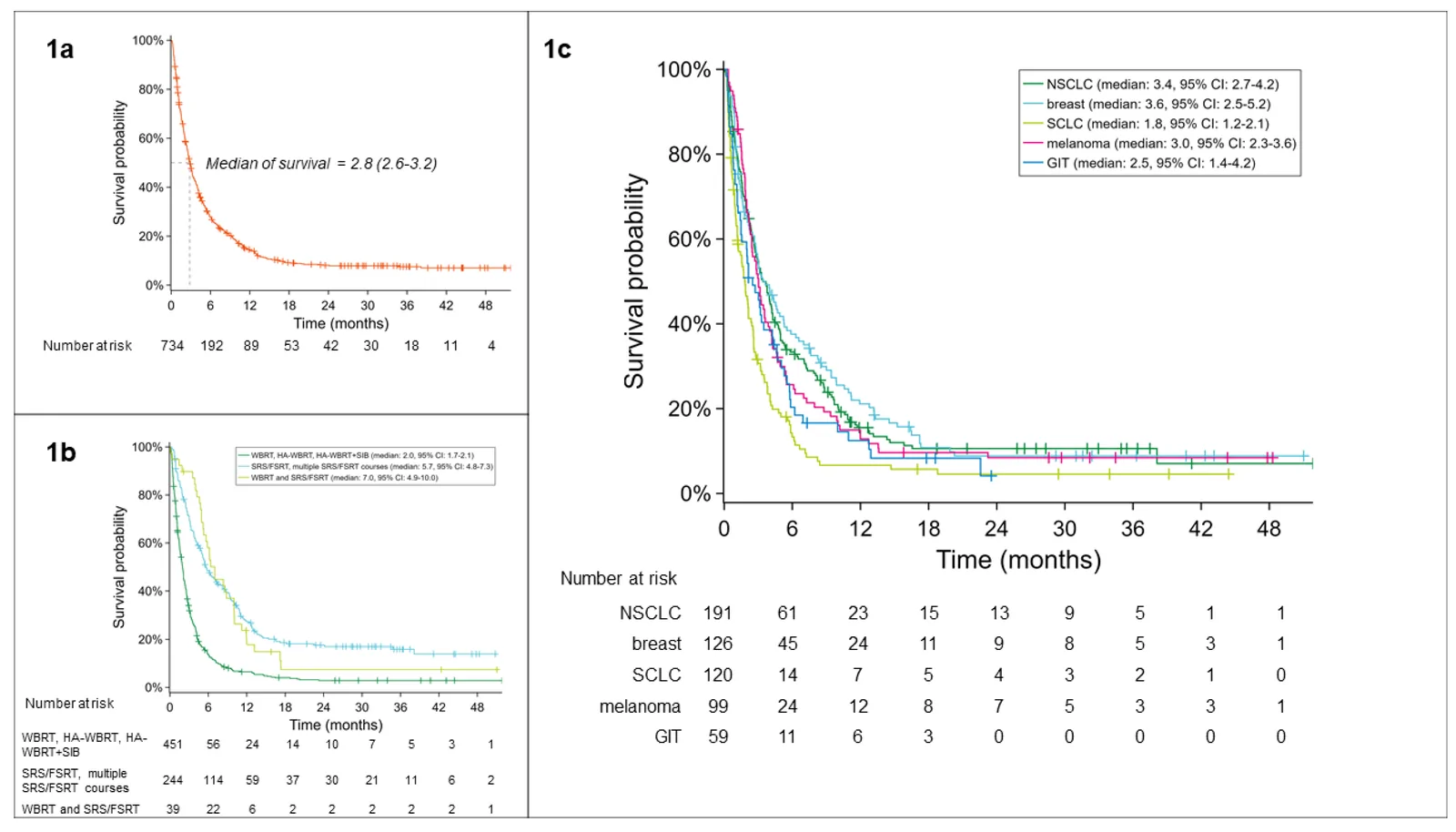
(**a**) Overall survival for the whole cohort, (**b**) overall survival according to type of radiotherapy, (**c**) overall survival according to type of primary cancer for five most common primary sites.

Median overall survival after RT of the whole set of patients was estimated to be 2.8 (2.6–3.2) months as summarized in Figure 1a. Median overall survival after radiotherapy according to the type of radiotherapy technique used was 2, 5.7 and 7 months for patients in group 1 (WBRT and WBRT modifications), group 2 (FSRT and SRS) and group 3 (WBRT + SRS/FSRT) respectively (Figure 1b).

Median OS according to tumor type was estimated with the longest median OS for breast cancer at 3.6 (2.5–5.2) months as summarized in Figure 1c. Table 2 shows a further subdivision of median OS for five most common primary diagnoses by radiotherapy type. In this case, breast cancer patients with single/repeated SRS of FSRT had 12.8 (7.5–17.2) months median OS and patients with SRS/FSRT and WBRT had a median OS of 11.9 (5.3–17.3) months.

Median overall survival for patients with NSCLC was 3.4 months (2.7–4.2 months), for WBRT group patients it was 2.1 (1.5–2.6) months, for single/multiple stereotactic irradiation it was 5.8 (3.9–8.7) months and for the combination of WBRT and stereotactic combination radiotherapy it was 6.2 months. The other three most common diagnoses, SCLC, melanoma and gastrointestinal malignancies, are listed in Table 2.

Median follow-up was 2.8 (0.1–51.8) months. Median follow-up estimated by the reverse KM method was 31.0 (28.3–34.5) months.

### 3.3. RT Techniques and Regimens During 2017–2023

**Figure 2:**
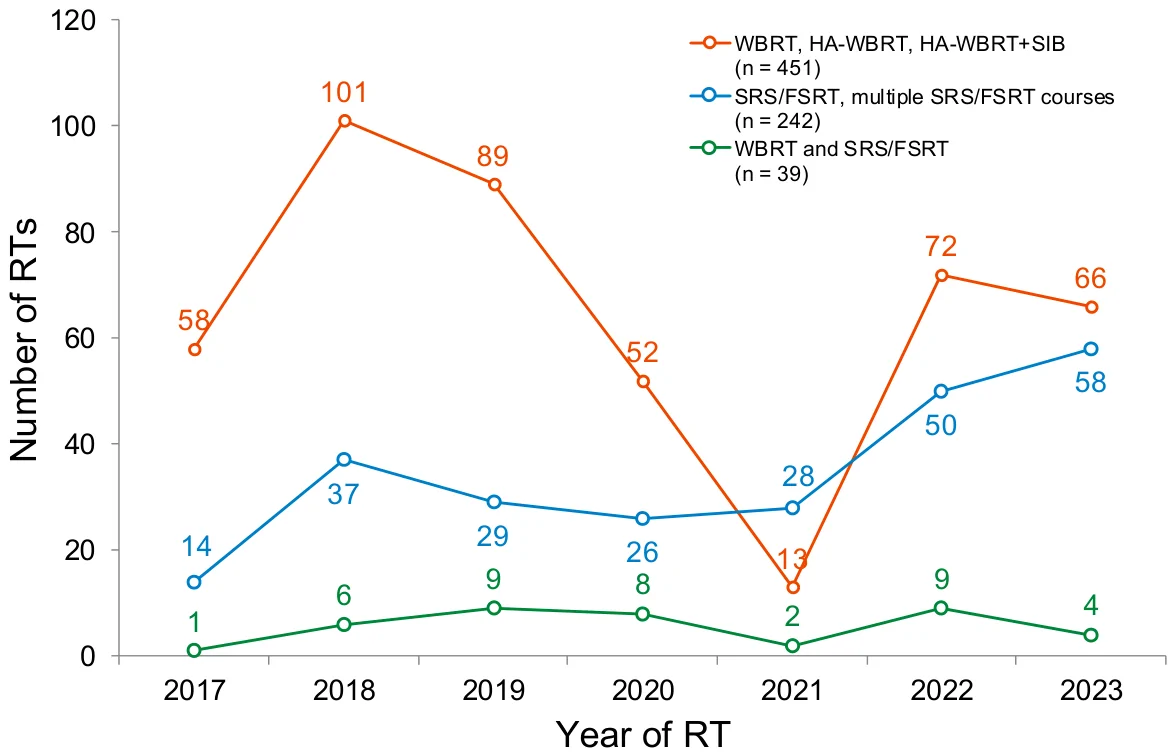
Numbers of irradiated patients during 2017–2023 distinguished by radiotherapy type. Abbreviations: WBRT: whole-brain radiotherapy; HA-WBRT: whole-brain radiotherapy—hippocampal avoidance; HA-WBRT-SIB: whole-brain radiotherapy—hippocampal avoidance with simultaneous integrated boost; FSRT: fractionated stereotactic radiotherapy; SRS: stereotactic radiosurgery; RT: radiotherapy.

Figure 2 shows the time trend of RT techniques utilized in our center. Years 2020 and 2021 show how the global pandemic of COVID-19 dramatically lowered the numbers of patients treated with radiotherapy for BM—a total number of 43 patients in 2021, compared to other years (usually 120–130 treated patients per year). Overall, a clear trend toward an increased integration of SRS/FSRT into routine radiotherapy practice was observed over the study period. The annual number of patients treated with SRS/FSRT from 2017 to 2023 was 14, 37, 29, 26, 28, 50, and 58, respectively. Despite some year-to-year variation, the overall number of patients treated with SRS/FSRT increased over time, with the highest number observed in 2023. Despite some year-to-year variation, the overall number of patients treated with SRS/FSRT increased over time, with the highest number observed in 2023.

For SRS, prescribed RT doses ranged from 18 to 21 Gy, with a single fraction of 21 Gy being the most commonly used regimen. In the FSRT group, the most frequently prescribed fractionation schedules were 3 × 9 Gy and 5 × 5 Gy. For WBRT and its variations, the most common RT fractionation schedule was 10 × 3 Gy.

## 4. Discussion

This article provides a survival analysis of 734 patients treated for brain metastases with practical focus on survival by tumor type and radiotherapy technique used. The number of patients treated for brain metastases has remained relatively stable over the years, although 2021 saw a lower number of patients (Figure 2). The decrease observed in 2021 may reflect the impact of the COVID-19 pandemic on the management of patients with brain metastases. During the pandemic, specific recommendations for patients with brain metastases emphasized minimizing hospital visits and supported the use of SRS in appropriately selected patients, while shorter WBRT schedules were preferred when WBRT was indicated [13]. In patients with a very limited prognosis, omission of radiotherapy in favor of supportive care was also considered appropriate. These pandemic-related changes in patient selection and treatment strategies may have contributed to the lower number of patients treated in our cohort in 2021. Radiotherapy constitutes an important component of palliative treatment for BM. Over the years, modern radiotherapy techniques have gradually been introduced into routine clinical practice. A few large retrospective analyses have systematically described baseline patient characteristics—for instance, Berghoff and colleagues analyzed a real-life cohort of 2419 patients treated at the Medical University of Vienna from 1990 to 2011, providing detailed descriptive statistics on demographic and disease-specific features across primary tumor types [14]. Our findings of tumor-type distribution compared to this analysis for the most common diagnoses are: lung cancer 42.3% vs. 43.2%, breast cancer 17.2% vs. 15.7%, melanoma 13.5% vs. 16.4%., and RCC 6.8% vs. 9.1%. Shifts might be caused by more accessible MRI imaging and by systemic treatment advances over time.

An analysis by Koide et al. described annual changes in RT techniques in 800 brain metastases patients treated during 2016–2021 [15]. A total of 597 patients (74.6%) underwent RT: 331 (41.4%) received SRS, 240 (30.0%) received WBRT and 26 (3.3%) underwent surgery plus radiotherapy. WBRT alone decreased significantly from the baseline year (28.7–18.8%), but SRS increased with or without systemic therapy (33.4–42.8%). Such annual trends—especially an increased use of SRS/FSRT—are noticeable in our cohort as well. These findings align with current knowledge on the quality of life of patients with brain metastases, particularly regarding efforts to preserve neurocognitive function [16].

Beyond the technical possibilities for cognitive preservation, pharmacotherapeutic options should also be mentioned, particularly the use of memantine [17]. The phase III NRG Oncology CC001 trial demonstrated that hippocampal avoidance during whole-brain radiotherapy combined with memantine significantly preserves cognitive function compared with standard whole-brain radiotherapy plus memantine [18].

We acknowledge several limitations of our study. First, the number of metastases per patient was not consistently reported, particularly for patients treated in earlier years with multiple brain metastases, limiting the completeness of these data. Systemic treatment and specific histology subtypes were not included in the analysis as our primary focus was on RT techniques. As a retrospective study, inherent limitations exist; however, the retrospective nature of the study reflects real-world practice. Selection bias favored patients who were considered eligible for stereotactic radiotherapy mostly due to their better performance status and control of the primary tumor.

### Future Perspectives

Looking ahead, further development of multidisciplinary management for patients with BM can be expected, especially in terms of optimizing the sequencing of therapeutic methods. Ongoing studies are evaluating the benefit of preoperative, rather than the currently standard postoperative, radiotherapy. Further development of new targeted drugs with CNS activity can also be anticipated; some are already being used in clinical practice, particularly for patients with asymptomatic BM from breast, melanoma and NSCLC cancer. SRS/FSRT will be used more frequently, reflecting a shift from criteria based on the number and size of brain metastases toward total intracranial tumor volume [2].

The increasing use of stereotactic radiotherapy techniques, including reirradiation, particularly in combination with targeted therapies or immunotherapy, will increase the need for accurate differential diagnosis of post-radiotherapy MRI changes and reliable treatment-response assessment. Emerging tools such as artificial intelligence and radiomics may contribute to improved differentiation between treatment-related changes and tumor progression. At the same time, the increasing use of technically demanding approaches such as SRS and FSRT has implications for treatment planning, image guidance, quality assurance, staffing, and treatment capacity. Therefore, monitoring changes in radiotherapy practice and treatment patterns may provide valuable information for planning future radiotherapy resources, infrastructure, and technology upgrades.

## 5. Conclusions

Management of patients with brain metastases has evolved through the integration of multidisciplinary approaches, advances in radiotherapy techniques, and the introduction of targeted systemic therapies with central nervous system activity. The increasing use of SRS and FSRT reflects a shift toward more individualized treatment strategies with the aim of achieving affective intracranial disease control while minimizing treatment-related toxicity and preserving quality of life and neurocognitive function. A clearly identifiable trend is a shift away from WBRT toward an increased use of targeted stereotactic irradiation techniques for brain lesions across various primary sites. However, the choice of treatment modality remains driven primarily by the oncological diagnosis and overall clinical context rather than by the radiotherapy technique itself. SRS and FSRT should not be regarded as replacements for WBRT, but rather as important components of an individualized multidisciplinary treatment strategy. In patients with a limited intracranial tumor burden and an appropriate clinical condition, stereotactic treatment may provide effective local control while avoiding some of the potential adverse effects associated with whole-brain irradiation. Conversely, WBRT or other approaches may remain appropriate in patients with extensive intracranial disease, unfavorable prognostic factors, or specific clinical circumstances in which focal treatment alone is unlikely to provide adequate disease control.

## Figures and Tables

**Table 1:** Description of basic patient characteristics.

	Total (n = 734)	WBRT, HA-WBRT, HA-WBRT-SIB (n = 451)	SRS/FSRT, Multiple SRS/FSRT Courses (n = 244)	WBRT and SRS/FSRT (n = 39)
Age (median, with min-max in brackets)	65.2 (23.8–86.7)	65.1 (23.8–86.7)	65.9 (28.3–84.8)	59.1 (34.6–85.9)
Sex (woman)	371 (50.5%)	228 (50.6%)	120 (49.2%)	23 (59.0%)
Primum (NSCLC)	191 (26.0%)	98 (21.7%)	84 (34.4%)	9 (23.1%)
Primum (breast)	126 (17.2%)	92 (20.4%)	23 (9.4%)	11 (28.2%)
Primum (SCLC)	120 (16.3%)	108 (23.9%)	11 (4.5%)	1 (2.6%)
Primum (melanoma)	99 (13.5%)	55 (12.2%)	34 (13.9%)	10 (25.6%)
Primum (GIT)	59 (8.0%)	29 (6.4%)	28 (11.5%)	2 (5.1%)
Primum (kidney)	50 (6.8%)	20 (4.4%)	27 (11.1%)	3 (7.7%)
Primum (ovaria)	12 (1.6%)	5 (1.1%)	6 (2.5%)	1 (2.6%)
Primum (other)	77 (10.5%)	44 (9.8%)	31 (12.7%)	2 (5.1%)

Abbreviations: WBRT: whole-brain radiotherapy; HA-WBRT: whole-brain radiotherapy—hippocampal avoidance; HA-WBRT-SIB: whole-brain radiotherapy—hippocampal avoidance with simultaneous integrated boost; FSRT: fractionated stereotactic radiotherapy; SRS: stereotactic radiosurgery; SCLC: small-cell lung carcinoma, NSCLC: non-small-cell lung carcinoma, GIT: gastrointestinal tumor.

**Table 2:** Median of survival with 95% confidence intervals according to type of primary cancer and type of radiotherapy.

	WBRT, HA-WBRT, HA-WBRT-SIB	SRS/FSRT, Multiple SRS/FSRT Courses	WBRT and SRS/FSRT
NSCLC	2.1 (1.5–2.6), n = 98	5.8 (3.9–8.7), n = 84	6.2 (0.0–10.1), n = 9
breast	2.7 (1.5–3.0), n = 92	12.8 (7.5–17.2), n = 23	11.9 (5.3–17.3), n = 11
SCLC	1.8 (1.1–2.4), n = 108	1.9 (0.9–4.2), n = 11	N/A
melanoma	2.2 (1.5–2.8), n = 55	5.4 (2.5–12.0), n = 34	4.6 (0.0–10.0), n = 10
GIT	1.2 (0.7–2.0), n = 29	5.5 (3.1–10.0), n = 28	5.8 (2.1–5.8), n = 2

Abbreviations: WBRT: whole-brain radiotherapy; HA-WBRT: whole-brain radiotherapy—hippocampal avoidance; HA-WBRT-SIB: whole-brain radiotherapy—hippocampal avoidance with simultaneous integrated boost; FSRT: fractionated stereotactic radiotherapy; SRS: stereotactic radiosurgery; SCLC: small-cell lung carcinoma, NSCLC: non-small-cell lung carcinoma, GIT: gastrointestinal tumor.

## Data Availability

The datasets are available from the corresponding author upon reasonable request.
